# How to estimate the sarcomere size based on oblique sections of skeletal muscle

**DOI:** 10.1111/joa.13892

**Published:** 2023-05-27

**Authors:** Federico Boschi

**Affiliations:** ^1^ Department of Engineering of Innovation Medicine University of Verona Verona Italy

**Keywords:** biopsy, MATLAB, sarcomere, skeletal muscle, Transmission Electron Microscopy

## Abstract

Ultrastructural analysis of muscular biopsy is based on images of longitudinal sections of the fibers. Sometimes, due to experimental limitations, the resulting sections are instead oblique, and no accurate morphological information can be extracted with standard analysis methods. Thus, the biopsy is performed again, but this is too invasive and time‐consuming. In this study, we focused our attention on the sarcomere's shape and we investigated which is the structural information that can be obtained from oblique sections. A routine was written in MATLAB to allow the visualization of how a sarcomere's section appears in ultrastructural images obtained by Transmission Electron Microscopy (TEM) at different secant angles. The routine was used also to analyze the intersection between a cylinder and a plane to show how the Z‐bands and M‐line lengths vary at different secant angles. Moreover, we explored how to calculate sarcomere's radius and length as well as the secant angle from ultrastructural images, based only on geometrical considerations (Pythagorean theorem and trigonometric functions). The equations to calculate these parameters starting from ultrastructural image measurements were found. Noteworthy, to obtain the real sarcomere length in quasi‐longitudinal sections, a small correction in the standard procedure is needed and highlighted in the text. In conclusion, even non‐longitudinal sections of skeletal muscles can be used to extrapolate morphological information of sarcomeres, which are important parameters for diagnostic purposes.

## Graphical abstract

The ultrastructural muscular morphology was investigated developing a computed model of the sarcomere. Using measurements done on virtual ultrastructural images and geometrical considerations, it is possible to calculate the sarcomere's radius and length, the Z‐bands’ length and the secant angle in muscle oblique sections.

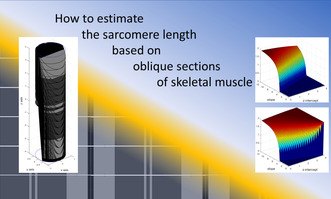

## 1 INTRODUCTION

Muscle biopsy is an important diagnostic tool for the study of neuromuscular pathologies (Cotta et al., 2021). In particular, it allows for distinguishing between neuropathies (affections of the nerves, which innervate the muscle) and myopathies (affections of the muscle tissue itself) (Jungbluth et al., 2018; Mercier et al., 2016). The complete diagnostic approach integrates data from different techniques including histochemical analysis, immunofluorescent microscopy, Transmission Electron Microscopy (TEM), and molecular studies. TEM is often used to investigate the muscle's ultrastructure, in particular shapes and dimensions of blood vessels, sarcolemma, nuclei, mitochondria, myofibrils, and sarcomeres (Cotta et al., 2021).

Skeletal muscles are composed of tubular muscle cells (myofibers) containing tubular myofibrils which consist of many consecutive sarcomeres (Henderson, Gomez, Novak, Mi‐Mi, & Gregorio, 2017). They form the basic units of the contractile apparatus (Squire, 1997). In TEM images, the sarcomeric architecture appears with several differently electron‐dense regions, each area resulting composed of many filaments connected with the peripheral areas, the so‐called Z‐disks, or within the central region, the M‐band, which divides into two specular halves the sarcomere (Pinotsis, Abrusci, Djinovic –Carugo, & Wilmanns, 2009). In particular, the different electron densities of the sarcomere in TEM images, due to the presence of thick and thin filaments of myosin and actin respectively, reveal the division of sarcomeres into different regions. Beyond the Z‐disk or Z‐line, limiting each sarcomere, there is the I‐band, surrounding the Z‐line, composed of thin filaments. Following the I‐band there is the A‐band containing both thick and thin filaments. Within the A‐band, there is the H‐zone composed of thick filaments and within the H‐zone, there is the M‐line that is in the middle of the sarcomere (Hu, Ackermann, & Kontrogianni‐Konstantopoulos, 2015; Lange, Ehler, & Gautel, 2006).

In a previous study, the parameters characterizing the sarcomere and myofibril arrangement were defined and a semi‐automatic routine to measure the parameters on ultrastructural (TEM) images was presented (Cisterna, Malatesta, Zancanaro, & Boschi, 2021). The routine was tested on skeletal muscle samples of trisomic (Down syndrome) and euploid mouse models, showing differences in sarcomere shape/arrangement in health and disease, confirming and expanding the results obtained by Cisterna, Sobolev, Costanzo, Malatesta, and Zancanaro (2020). Apart from Down syndrome, sarcomere size can be affected in different other disease. For instance, sarcomere length could be increased in diabetes, altering ventricular function (Isola et al., 2021).

Generally, morphological pattern in ultrastructural images is evaluated on sections obtained by cutting the myofibers with a plane parallel to the fibers’ axis (longitudinal sections). In histological practice, the sections are not always longitudinal and redoing the sampling might be invasive and time‐consuming. Here, which are the morphological information obtainable from virtual sections of muscle fibers, was investigated. The study was focused on the sarcomere's radius and length, and the angle formed by the secant plane with the sarcomere's axis. A routine simulating how a sarcomere appears when it is cut at different angles was written in MATLAB. Hypothesizing that the sarcomere's shape can be considered as a cylinder and using the Pythagorean theorem and trigonometrical equations, has been found that it is possible to obtain the cylinder's size (the sarcomere dimensions) starting from the shape and dimensions of the cylindric sections (i.e., the intersection of a cylinder's surface with a plane) for many different secant angles (oblique sections). Finally, using the same routine, how the Z‐band and M‐line lengths change, varying the secant angles, is described. This study refers principally to the skeletal muscles; in fact, cardiomyocytes have a general shape that cannot be related to a cylinder.

## 2 METHODS

### 2.1 Hypothesis and geometrical parameters

**FIGURE 1:**
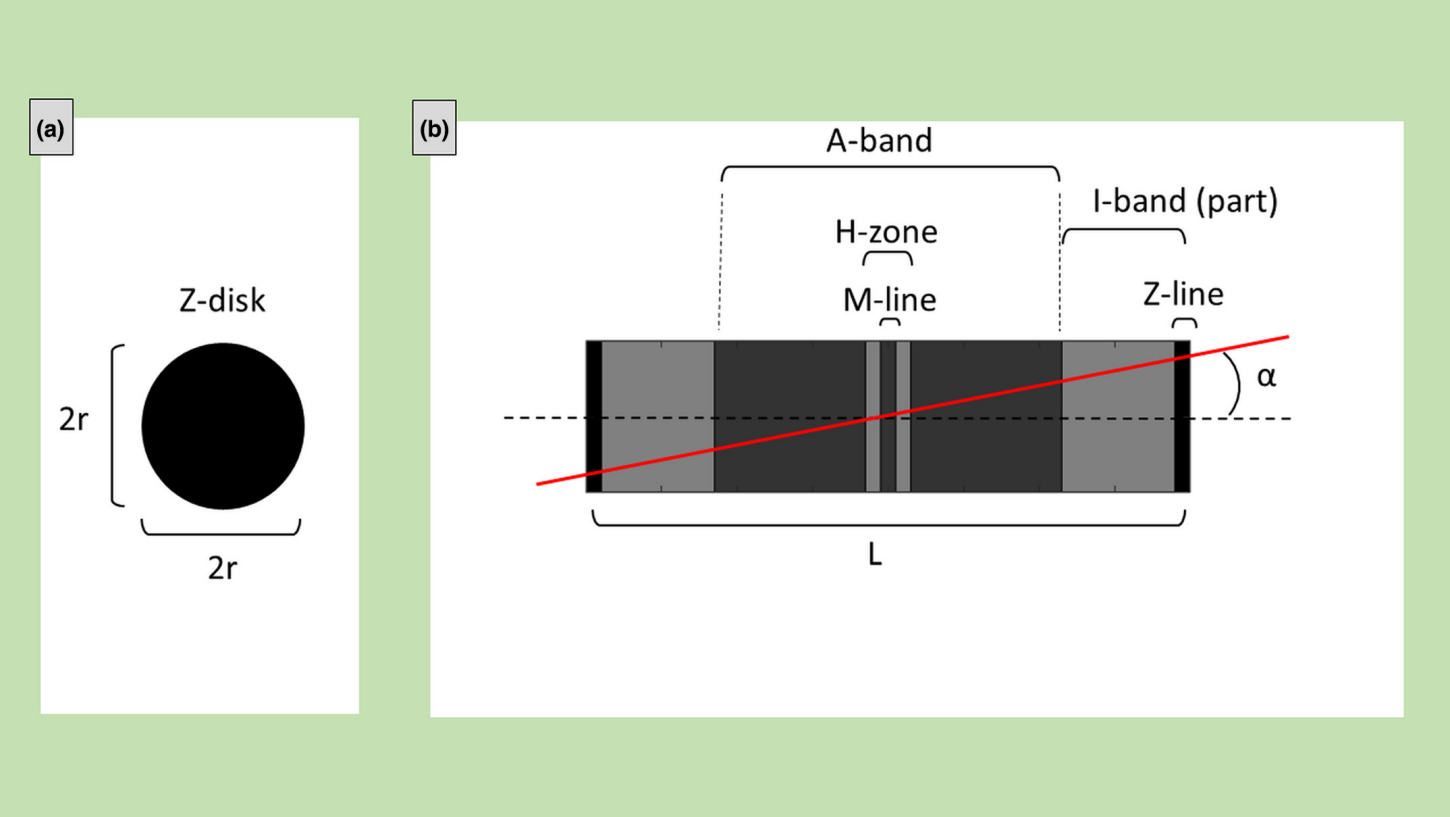
Axial (a) and lateral view (b) of a sarcomere represented as a cylinder with radius (*r*), length (*L*), and cut with a plane (red line) forming an angle (*α*) with the sarcomere axis. The principal sarcomere features, visible in ultrastructural images, the A‐band, I‐band (which involves also the adjacent sarcomere), M‐line, Z‐lines, and H‐zone, are also shown.

The sarcomere is considered here as a right circular cylinder (Figure 1) and many sarcomeres are aligned along the same axis to form a straight myofibril. The geometrical parameters of the sarcomere are: *L* = cylinder's length (i.e., sarcomere's length), *r* = cylinder's radius (half size of Z‐line length), *α* = secant angle (the angle between the plane and the cylinder's axis).

The intersection of the cylinder with a plane (secant plane), the cylindric section, can have a different shape (circular, ellipse or parallelogram, or others). The bases of the cylinder are the Z‐disks; the intersection of the cylinder with the plane passing in the center of the cylinder and parallel to the bases is the M‐line. Both Z‐disks and the M‐line are generally well visible in ultrastructural images due to the contrast with the paler surrounding areas and it aids the measurements.

### 2.2 MATLAB routine

A routine was written in MATLAB (R2018b version, Mathworks) to graphically visualize the sarcomere's sections at different secant angles. Using a Cartesian coordinate system (*x*, *y*, *z*), the sarcomere is represented by a right cylinder, centered on the origin, oriented along the *z*‐axis, with *r* = 1 and *L* = 8, from *z* = −4.0 to *z* = +4.0. We assumed here that the ratio *L/r* is equal to 8 as reported in the literature (Cisterna et al., 2021). The cylinder is subdivided into different regions with dimensions similar to the sarcomere's bands and colored with different gray shades similar to the ones observed in TEM images (Cisterna et al., 2021).

The sarcomere's regions are:

lower Z‐disk: height = 0.2, from *z* = −4.0 to *z* = −3.8, RGB color [0.0 0.0 0.0];
lower I‐band part: height = 1.5, from *z* = −3.8 to *z* = −2.3, RGB color [0.5 0.5 0.5];
lower A‐band part: height = 2.0, from *z* = −2.3 to *z* = −0.3, RGB color [0.2 0.2 0.2];
lower H‐zone part: height = 0.2, from *z* = −0.3 to *z* = −0.1, RGB color [0.5 0.5 0.5];
M‐line: height = 0.2, from *z* = −0.1 to *z* = +0.1, RGB color [0.2 0.2 0.2];
upper H‐zone part: height = 0.2, from *z* = +0.1 to *z* = +0.3, RGB color [0.5 0.5 0.5];
upper A‐band part: height = 2.0, from *z* = +0.3 to *z* = +2.3, RGB color [0.2 0.2 0.2];
upper I‐band part: height = 1.5, from *z* = +2.3 to *z* = +3.8, RGB color [0.5 0.5 0.5];
upper Z‐disk: height = 0.2, from *z* = +3.8 to *z* = +4.0, RGB color [0.0 0.0 0.0].

In order to characterize the secant plane, we considered the sheaf of planes that have the same common line represented by the *x*‐axis, which has the equation *z* = m*y + *c*, where *m* is the slope and *c* is the *z*‐intercept. The parameters *m* and *c* define the secant plane and, in the routine, *m* can vary in the [0, +12] range and *c* in the [0, +4] range.

In addition, the routine allows measuring the length of the segments of the cylindric section when the plane intersects the bases of the cylinder itself and the M‐line (Z‐line length and M‐line length, respectively) calculating the distance between two points in the coordinate system.

## 3 RESULTS

### 3.1 Longitudinal sections

In longitudinal sections (*α* = 0°), the sarcomere's length and the Z‐disks size can be measured directly on the images. Ideally, all the sarcomeres composing the same myofibril are visible in TEM images.

### 3.2 Almost longitudinal sections

**FIGURE 2:**
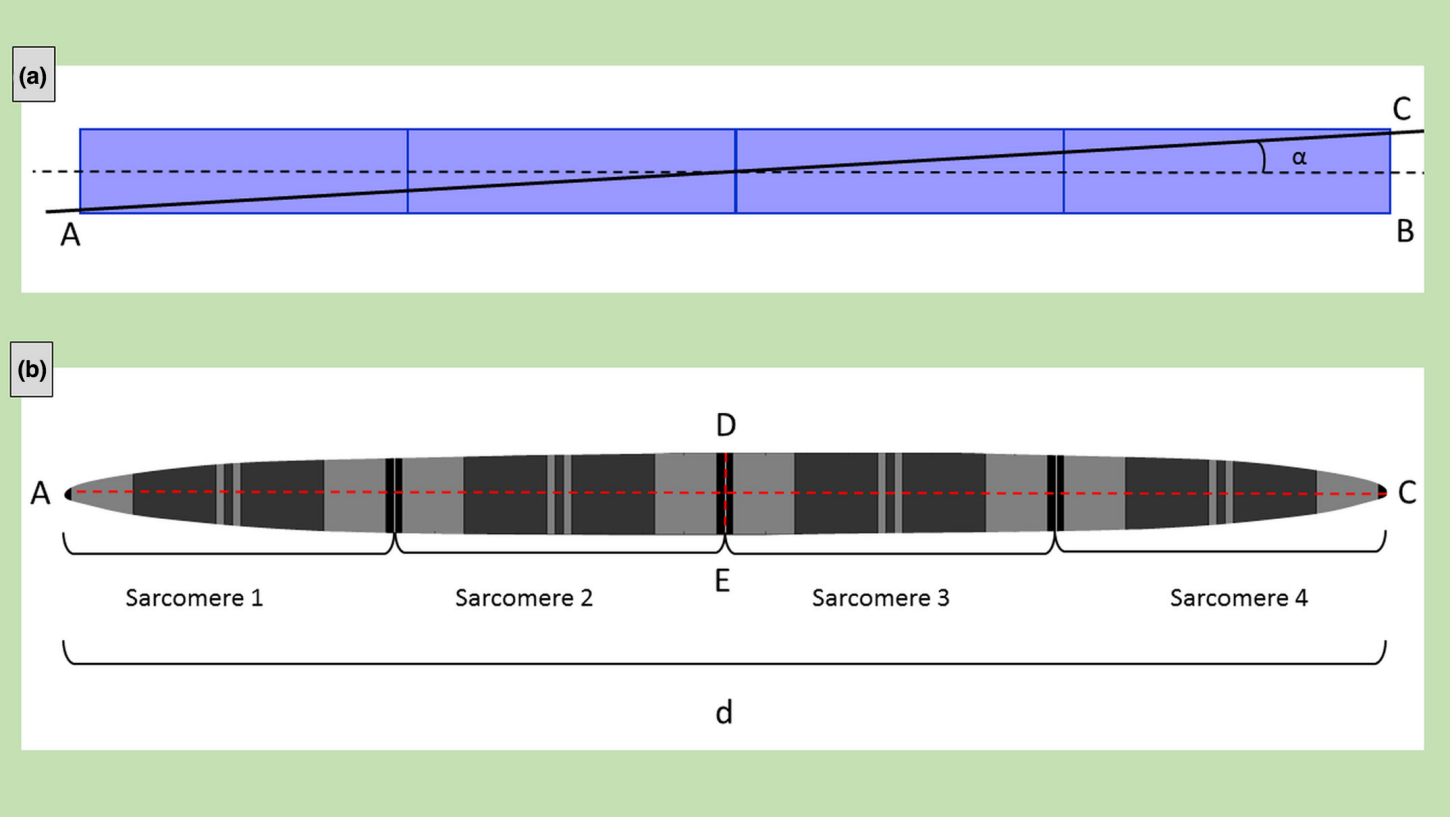
Example of almost longitudinal section. Schematic representation of four aligned sarcomeres of the same myofibril (a). The myofibril is cut with a plane (AC, black line) forming an angle (*α*) with the myofibril axis. Simulated representation of the myofibril section (b) as visible in ultrastructural images.

In almost longitudinal section (*α* ~ 0°), a number *n* of aligned sarcomere belonging to the same myofibril are visible in ultrastructural images. Figure 2 represents four (*n* = 4) aligned sarcomeres and the secant plane (Figure 2a) and how the ultrastructural section (Figure 2b) appears when the secant plane is tangent to the lower part of the Z‐disk of the first sarcomere on the left and to the upper part of the Z‐disk of the last sarcomere on the right.

Measuring the distances AC and DE on the ultrastructural image it is possible to obtain *r*, *L*, and *α*.

Being *DE* = 2*r*, it follows that *r* = *DE/2*. Indicating *AC* = *d* and using the Pythagorean theorem, *L* can be determined as:

(1) $$\;L=\frac{\sqrt{{\mathrm{AC}}^{2}-{\mathrm{DE}}^{2}}}{n}=\frac{\sqrt{d^{2}-4r^{2}}}{n}$$

It is worth noting that *L* is not equal to *AC/n* or *d/n*, or, in fact, the distance between two consecutive Z‐bands measured in the ultrastructural images (the apparent sarcomere's length) is not equal to the real sarcomere length (being the hypotenuse and a cathetus of a right triangle, respectively).

More precisely, the real and the apparent lengths are related by the following equation:

(2) $$L=L_{\text{apparent}}\;\cos\;\alpha$$

Clearly, the greater the number of visible aligned sarcomeres in the ultrastructural section, the lower the difference between the apparent and the real lengths.

Using the trigonometric functions, *α* can be calculated as follow (being *BC* = *DE*):

(3) $$\;\alpha =\sin^{-1}\;\frac{\mathrm{DE}}{\mathrm{AC}}=\sin^{-1}\;\frac{2r}{d}$$

Knowing *L* from Equation 1, *α* can be obtained also from:

(4) $$\;\alpha =\tan^{-1}\;\frac{\mathrm{CB}}{\mathrm{AB}}=\tan^{-1}\;\frac{2r}{\mathrm{nL}}$$

Considering that, morphological studies on mouse skeletal muscle reported that in healthy conditions sarcomeres are cylinder with length approximately 4‐fold longer than the Z‐band length (Cisterna et al., 2021), *r* is approximately 1/8 of *L*, and thus:

(5) $$\;\alpha =\tan^{-1}\;\frac{1}{4L}$$

Equation 5 gives *α* = 14.04°, 7.13°, 4.76°, 3.58°, 2.86°, 2.39°, 2.05°, and 1.79°, when *n* = 1, 2, 3, 4, 5, 6, 7, or 8 sarcomeres are visible along the same myofibril, respectively. In the example shown in Figure 2, *α* is 3.58°, being *n* equal to 4.

For simplicity, only symmetrical conditions (secant plane tangent to both Z‐disks) are considered in this sub‐section. In case of asymmetry, slight corrections can be done using the same geometrical considerations.

### 3.3 Oblique sections with two visible Z‐lines

In oblique sections, if *α* is low, many sarcomeres are intersected by the plane and the situation is the same as described in the previous sub‐section. Instead, increasing *α*, both the bases of the cylinder (the Z‐disks) can be intersected by the secant plane and two Z‐lines are visible in the images.

**FIGURE 3:**
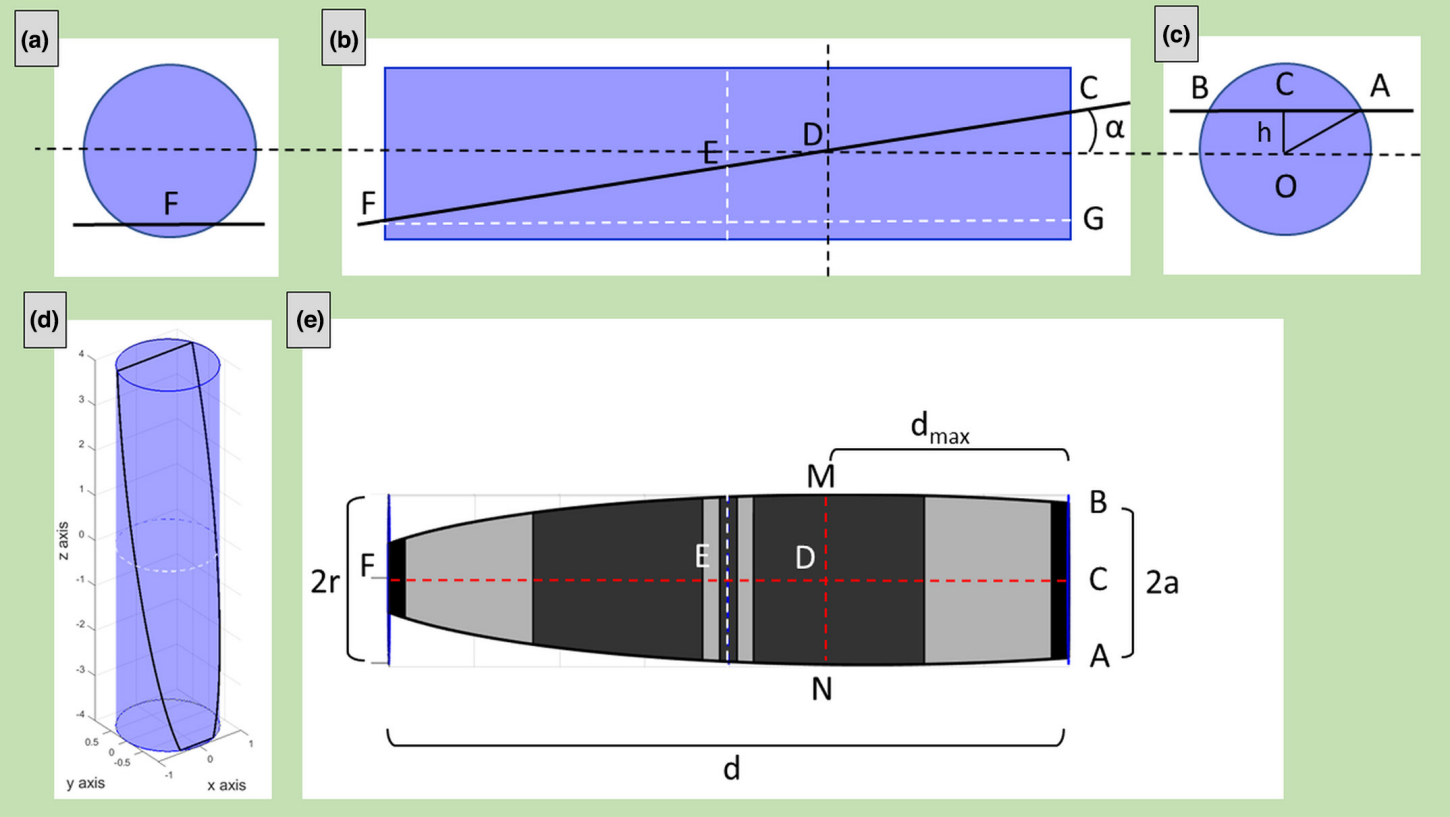
Example of oblique section with two visible Z‐lines. The secant plane is passing through both the sarcomere's Z‐disks. Three different point of views of the cylinder (a, b, c); the cylinder with the cylindric section (black line) obtained with plane slope *m* = 6 and *z*‐intercept *c* = 1.5 (d) and the corresponding sarcomere's ultrastructural section (e).

To obtain *r*, *L*, and *α*, the distances *MN = 2r*, *AB = 2a*, CF = *d*, and *CD* = *d*_*max*_, need to be measured on ultrastructural images (Figure 3). *d* is the distance between the two Z‐lines (apparent sarcomere's length) and *d*_*max*_ is the distance, measured on the cylindrical axis, between one Z‐line and the point where the width of the cylindrical section is maximum. The radius *r* is *MN/2*.

Looking at Figure 3c, the distance *OC = h* can be evaluated with the Pythagorean theorem:

$$h=\sqrt{{\mathrm{OA}}^{2}-{\mathrm{AC}}^{2}}=\sqrt{r^{2}-a^{2}}$$

but *h* is also related to *d*_*max*_ and *α* by the following equation (Figure 3b):

$$\;h=\mathrm{CD}\sin\alpha =d_{\max}\sin\alpha .$$

Thus, *α* can be evaluated as:

$$\alpha =\sin^{-1}\frac{h}{d_{\max}}$$

Moreover, in the right triangle CFG (Figure 3b), the following relationship is valid:

GF=CFcosα

which leads to the final result:

L=dcosa

### 3.4 Oblique sections with only one visible Z‐line …

In oblique section with only one visible Z‐line, two different scenarios are possible if the M‐line is visible or not.

#### 3.4.1 And visible M‐line

**FIGURE 4:**
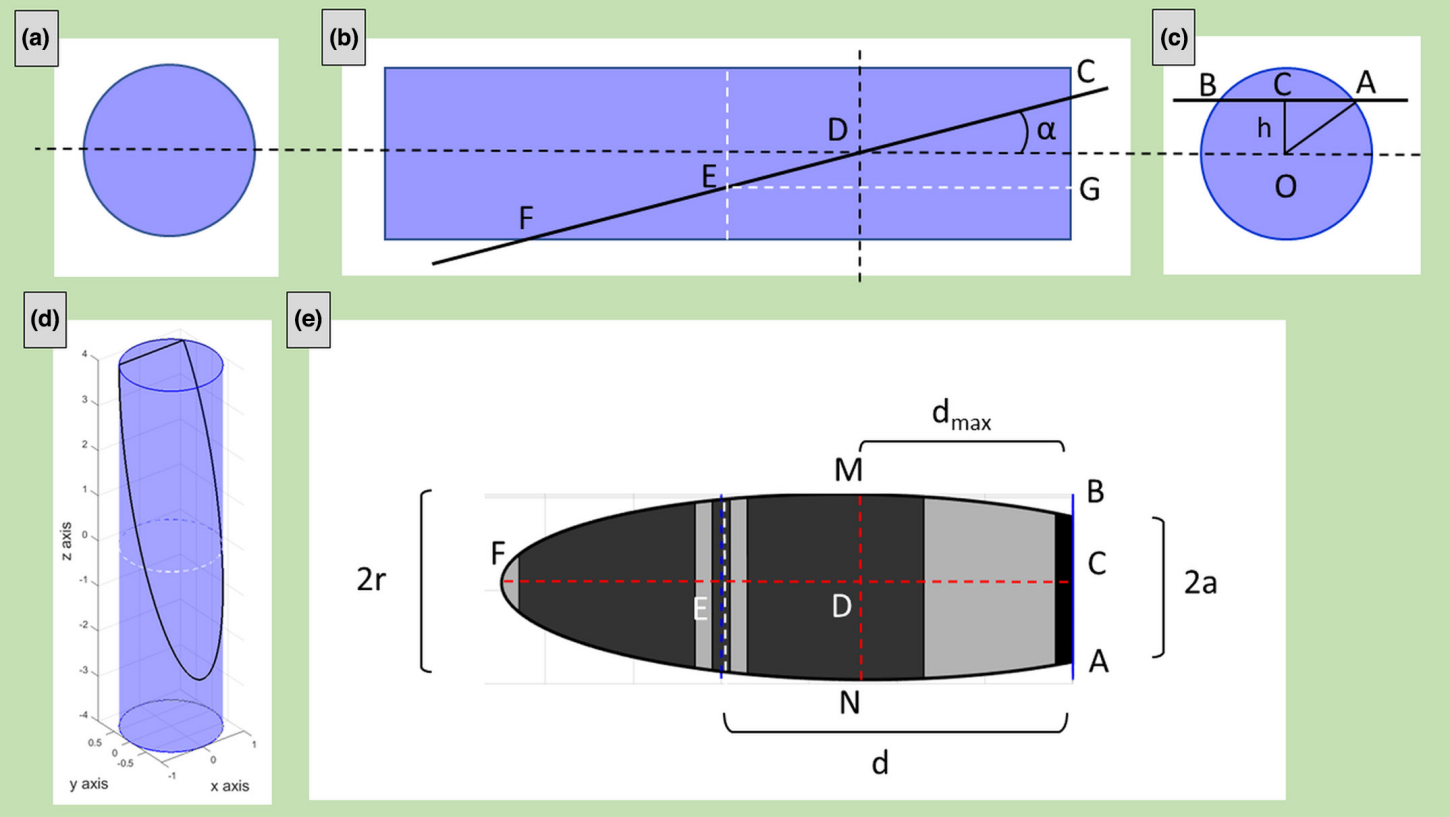
Example of oblique section with only one visible Z‐line and visible M‐line. The secant plane is passing through only one sarcomere's Z band (but is passing also through the M line). Three different point of views of the cylinder (a, b, c); the cylinder with the cylindric section (black line) obtained with plane slope *m* = 4 and *z*‐intercept *c* = 1.5 (d) and the corresponding sarcomere's ultrastructural section (e).

In this case, the plane crosses only one base (only one Z‐disk) but intersects the M‐line. In the ultrastructural images, the missing information regarding the total apparent sarcomere's length is substituted by the measure of the half apparent length *d* (Figure 4) which represents the distance between the Z‐line and the M‐line.

To calculate *r*, *L* and *α*, it is necessary to measure on TEM images the distances *MN = 2r*, *AB = 2a*, *d*, and *d*_*max*_ (Figure 4). Thus, *r* = *MN/2*.

As done in the previous sub‐section, *α* can be obtained from:

$$\alpha =\sin^{-1}\frac{h}{d_{\max}}$$

Moreover, CEG is a right triangle (Figure 4b), thus:

GE=CEcosα

Substituting *L* and *d*:

$$\frac{L}{2}=d\cos a$$

and finally:

L=2dcosa

#### 3.4.2 Without visible M‐line

When only one Z‐disk is intersected by the secant plane and the M‐line is no longer visible, only *r* and *α* are measurable.

**FIGURE 5:**
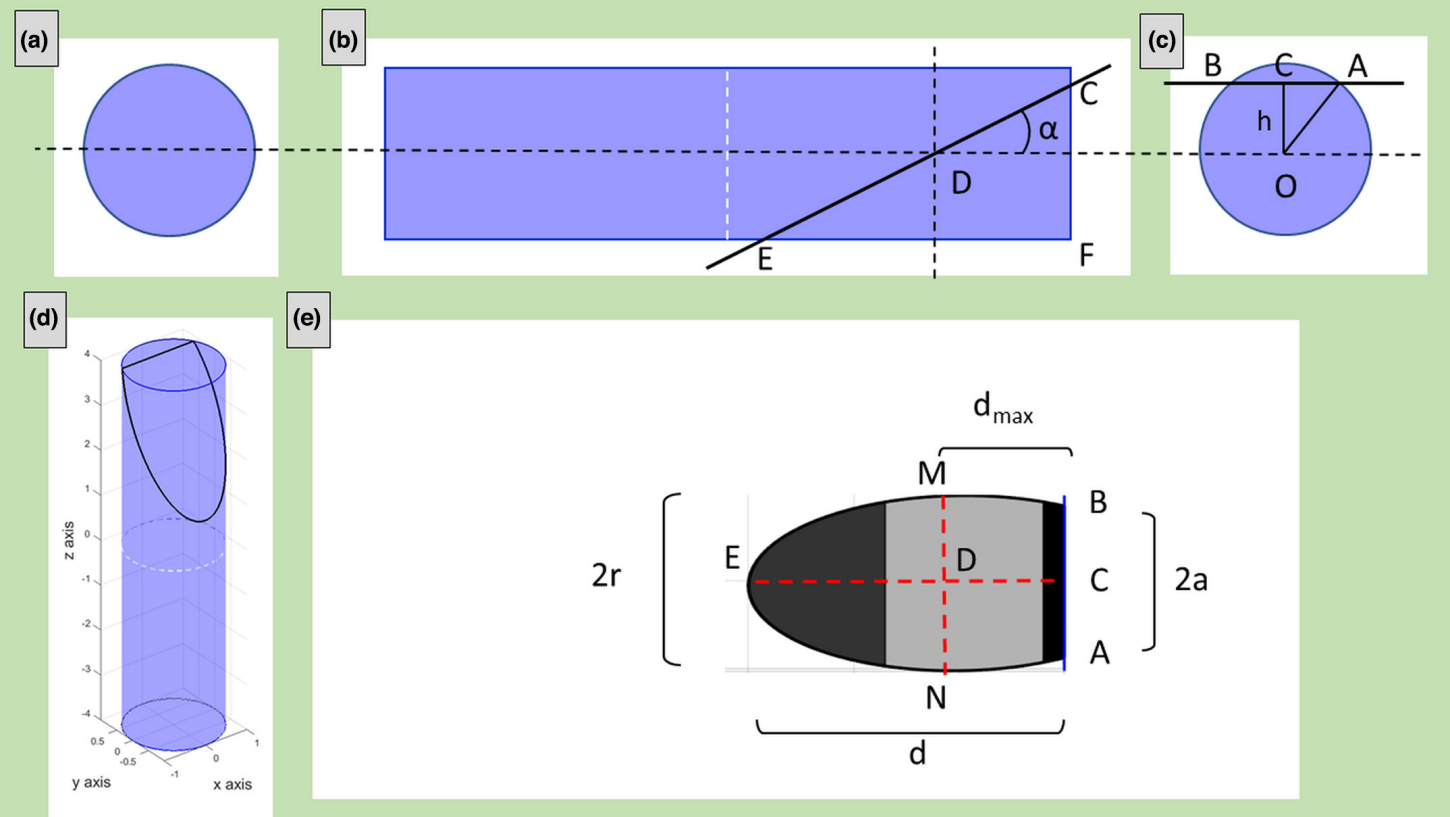
Example of oblique section with only one visible Z‐line and no visible M‐line. The secant plane is passing through only one sarcomere’ Z band (without passing through the M line). Three different point of views of the cylinder (a, b, c); the cylinder with the cylindric section (black line) obtained with plane slope *m* = 2 and *z*‐intercept *c* = 3.0 (d) and the corresponding sarcomere's ultrastructural section (e).

Measuring *MN = 2r*, *CD = d*_*max*_ *and AB = 2a* (Figure 5) it is possible to calculate *r* and *α*. Again, *r = MN/2* and.

$$\alpha =\sin^{-1}\frac{h}{d_{\max}}$$

or, alternatively, looking at the right triangle CEF (Figure 5b),

$\alpha {=\sin}^{-1}\frac{r+h}{d}$, being more useful, because *d* is more easily measurable than *d*_*max*_.

### 3.5 Almost transversal section

**FIGURE 6:**
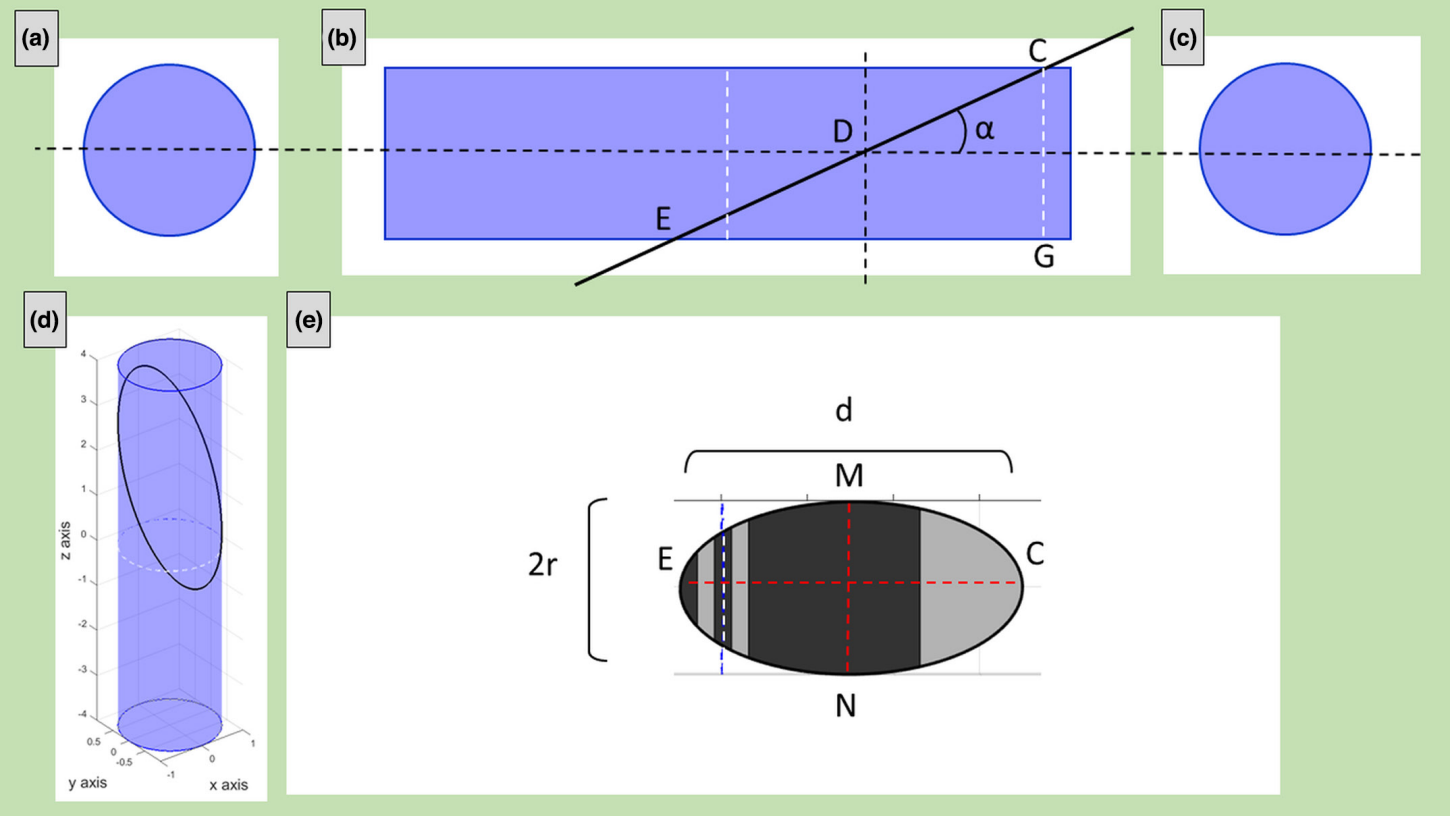
Example of almost transversal section. The secant plane is not passing through the sarcomere’ Z bands. Three different point of views of the cylinder (a, b, c); the cylinder with the cylindric section (black line) – which is an ellipse‐ obtained with plane slope *m* = 2 and *z*‐intercept *c* = 1.5 (d) and the corresponding sarcomere's ultrastructural section (e).

In almost transversal section, the secant plane could not intersect the cylinder's bases and the cylindric intersection is an ellipse. In the ultrastructural images, no Z‐lines are visible (Figure 6). It is no longer possible to know the length of the sarcomere, but it is still possible to measure *r* and *α*.

To do this, it is sufficient to measure *MN = 2R* and CF (Figure 6). Also, in this case *r = MN/2*.

In the right triangle, CEG (Figure 6b) the following relationship is valid:

CG=CEsinα

Then, *α* is:

$$\alpha =\sin^{-1}\frac{2r}{d}$$

### 3.6 Transversal section

In perfect transversal section (*α* = 90°) the cylindric section is a circle and the radius is the only obtainable parameter. It is sufficient to measure the diameter and halve the obtained value.

### 3.7 Summary

Table 1 shows the geometrical features that can be obtained from sections at different secant angles and the needed measurements on the ultrastructural images.

**TABLE 1:** Varying the secant angle *α* from 0° to 90° (1st column), i.e., from longitudinal to transversal section, the visible sarcomeres' features in ultrastructural images (2nd column), the shape of the cylindric section (3rd column), the sarcomeres' size (*r*, *L*) and secant angle (*α*, 4th, 5th and 6th columns, respectively) obtainable from the images are shown. In the 7th column the measures necessary to obtain the sarcomeres' morphological parameters are indicated.

	Sarcomere's visible features	Cylindric section	Radius *r*	Length *L*	Angle *α*	Measurements
Longitudinal section (*α* = 0°)	2 Z‐lines	rectangle	Yes	Yes	*α* = 0°	*r* and *L* are directly measurable
Almost longitudinal section	2 Z‐lines		Yes	Yes	Yes	*d, DE* (see Figure 2)
Oblique section	2 Z‐lines		Yes	Yes	Yes	*d, d*_*max*_, *AB* (see Figure 3)
Oblique section	1 Z‐line and M‐line		Yes	Yes	Yes	*d, d*_*max*_, *AB* (see Figure 4)
Oblique section	1 Z‐line		Yes	No	Yes	*MN, d*_*max*_, *AB* (see Figure 5)
Almost transversal section	No Z‐lines	Ellipse	Yes	No	Yes	*FC, MN* (see Figure 6)
Transversal section (*α* = 90°)	No Z‐lines	Circle	Yes	No	*α* = 90°	Diameter of the circle is directly measurable

### 3.8 Z‐lines and M‐line length measurement

**FIGURE 7:**
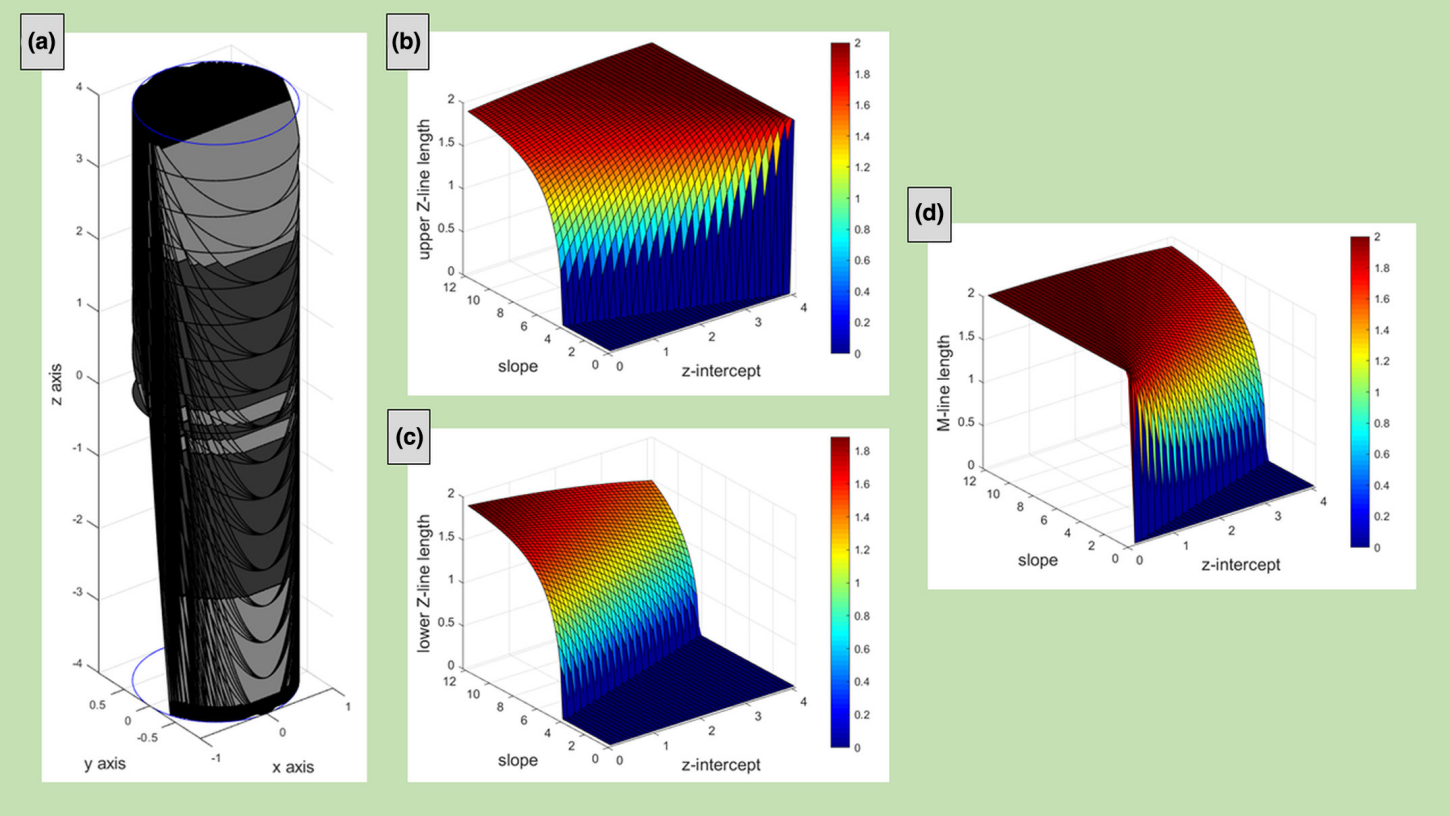
Cylinder's section obtained varying both the slope m of the sheaf of planes and the z‐intercept c. The parameters used are m in the range 0–12 with step width 1 and c in the range 0–4, with step width 0.5 (a). Upper Z‐line length (b), lower Z‐line length (c), and M‐line length (d) as function of the slope *m* and *z*‐intercept *c*.

The routine written in MATLAB shows the cylindric section and the appearance of the sarcomere cut by different planes. Figure 7a shows the cylinder's sections obtained varying the slope *m* from 0 to 12 with step width 1 and the z‐intercept *c* from 0 to 4 with 0.5 step width. The routine also measures the size of the linear segments (Z‐line length and M‐line length) when the Z‐disks and the M‐line of the sarcomere are intersected by the secant plane.

Figures 7b–d report the size of the upper Z‐line, lower Z‐line (upper and lower with respect to the *z*‐axis), and the M‐line length varying the slope and the z‐intercept of the plane. To better understand the figures, some particular cases are here illustrated. When *c* = 0 the upper Z‐line starts to be visible at *m* = 4 and increases with *m* to the limit value = 2 (Figure 7b). When *c* = 4 the Z‐line length is 2 for all the slopes (Figure 7b). Also, the lower Z‐line starts to be visible at *c* = 0 and *m* = 4, but its length decreases for all the slopes increasing the z‐intercept (Figure 7c).

Instead, the M‐line length is always = 2, for each slope, when *c* = 0 and is always 0 if *c* ≠ 0 e *m* = 0 (Figure 7d).

## 4 DISCUSSION

TEM ultrastructural analysis represents a meaningful tool for pathological neuromuscular investigation, giving information on the myofibril shape, mitochondrial structure, sarcolemmal, and sarcomere features (Burkholder & Lieber, 2001; McMillan & Eisenback, 2018; Picard, White, & Turnbull, 2013). It is generally conducted on muscular biopsy and the most informative ultrastructural images are considered those obtained from longitudinal sections of the fiber, thus oblique sections are often not considered for analysis.

In this work, only through geometrical considerations, we showed that it is possible to calculate the length and the radius of the sarcomeres as well as the inclination of the secant plane starting from ultrastructural images of skeletal muscle also in the case of oblique sections. Moreover, in this work, a digital model of a sarcomere useful to simulate how it appears in ultrastructural images at different secant angles was presented.

The hypothesis of an almost cylindrical shape on which the mathematical evaluations are based, is particularly true for skeletal muscle, in spite of cardiomyocytes having a shape that cannot be related to a cylinder. The skeletal muscle sarcomere features obtainable at different cut angles are explained in the text and the equations to obtain the results are reported. In addition, we showed that, even in not longitudinal perfectly sections, the length of the sarcomere is not the apparent distance between the two Z‐bands that confine it, but that distance must be corrected for the cosine of the secant angle (Equation 2).

In this study, we did not consider the case of longitudinal sections in which the cutting plane, parallel to the myofibril's axis, it is quite far from it. In this condition, the sarcomeres appear with a radius lower compared to those of the adjacent myofibrils, so the measurements of those sarcomeres are generally excluded from the analysis. In addition, it is important to mention that although sarcomere behavior in contraction it is considered to be uniform, the occurrence of sarcomere length nonuniformities is well known in the literature (Burkholder & Lieber, 2001; de Souza Leite & Rassier, 2020; Edman & Reggiani, 1984; Edman & Reggiani, 1987; Julian & Morgan, 1979; Ralston et al., 2008). Moreover, the size of the different types of myofibrils can be increased or reduced by the fixative reagents (Davidowitz, Rubinson, Jacoby, & Philips, 1996). In longitudinal sections, a high number of sarcomeres are visible along the same myofibril, and to increase the number of measurements, the analysis can be performed on the same myofibril and in the adjacent ones. Instead, in the case of oblique sections only one sarcomere (the cut one) is visible for each myofibril, so measurements should be made also on many adjacent myofibrils. Thus, a large number of measurements is necessary to reduce the effects of nonuniformities and to achieve statistical significance.

The proposed method is based on the manual extraction of the measurements. A completely automated software able to extract the same information on a large number of sarcomeres could be based on a fit between the real data and the shape of the theoretical cylindric section or on the Hough transform (Duda & Hart, 1972; Hart, 2009). The high contrast of Z‐bands and of the M‐line with respect to the surrounding areas can help the automated analysis.

Besides the length, the approach here presented might also be useful to calculate the radius of the sarcomere and the myofibrils diameter. This specific parameter has to be considered when comparing different muscles since the sarcomere size could vary among different muscles (Burkholder & Lieber, 2001; Schönbauer et al., 2011) and further the improper regulation of the sarcomere diameter could be a sign of myopathies (González‐Morales et al., 2019; Katti et al., 2022). For these reasons, it could be interesting to compare not only the mean sarcomere size but also the sarcomere size distribution of the data among different pathological conditions.

The proposed equations allow for comparing sarcomere lengths from different samples and species as well as in physiological and pathological conditions. Furthermore, in the case of the anisotropic effects of the fixation procedure on the sarcomere size (e.g., greater reduction in sarcomere length compared to the reduction in the radial direction) the proposed equations still work.

In conclusion, this work opens the possibility to consider extracting all possible information from ultrastructural images even in the case of not perfectly longitudinal sections. Next step could be the application of the presented equation on real TEM images. This approach could be considered a useful tool for skeletal muscle morphological analysis.

## FUNDING INFORMATION

Author FB declares that no funding was received for conducting this study.

## CONFLICT OF INTEREST STATEMENT

Author FB declares that he has no conflict of interest.

## Data Availability

The data that support the findings of this study are available from the corresponding author upon reasonable request.

## References

[joa13892-bib-0001] Burkholder, T. & Lieber, R. (2001) Sarcomere length operating range of vertebrate muscles during movement. Journal of Experimental Biology, 204, 1529–1536. 11296141 10.1242/jeb.204.9.1529

[joa13892-bib-0002] Cisterna, B. , Malatesta, M. , Zancanaro, C. & Boschi, F. (2021) A computational approach to quantitatively define sarcomere dimensions and arrangement in skeletal muscle. Computer Methods and Programs in Biomedicine, 211, 106437. 34624632 10.1016/j.cmpb.2021.106437

[joa13892-bib-0003] Cisterna, B. , Sobolev, A.P. , Costanzo, M. , Malatesta, M. & Zancanaro, C. (2020) Combined microscopic and metabolomic approach to characterize the skeletal muscle fiber of the Ts65Dn mouse, a model of down syndrome. Microscopy and Microanalysis, 26, 1014–1023. 32867866 10.1017/S143192762002437X

[joa13892-bib-0004] Cotta, A. , Carvalho, E. , da‐Cunha Junior, A.L. , Valicek, J. , Navarro, M.M. , Junior, S.B. et al. (2021) Muscle biopsy essential diagnostic advice for pathologists. Surgical Experimental Pathology, 4, 1–20.

[joa13892-bib-0005] Davidowitz, J. , Rubinson, K. , Jacoby, J. & Philips, G. (1996) Myofibril size variation along the length of extraocular muscle in rabbit and rat. I: orbital layer. Tissue & Cell, 28, 63–76. 8907727 10.1016/s0040-8166(96)80045-0

[joa13892-bib-0006] de Souza Leite, F. & Rassier, D.E. (2020) Sarcomere length nonuniformity and force regulation in myofibrils and sarcomeres. Biophysical Journal, 119, 2372–2377. 33217382 10.1016/j.bpj.2020.11.005 PMC7822744

[joa13892-bib-0007] Duda, R.O. & Hart, P.E. (1972) Use of the Hough transformation to detect lines and curves in pictures. Communications of the ACM, 15, 11–15.

[joa13892-bib-0008] Edman, K.A. & Reggiani, C. (1984) Redistribution of sarcomere length during isometric contraction of frog muscle fibres and its relation to tension creep. The Journal of Physiology, 351, 169–198. 6611407 10.1113/jphysiol.1984.sp015240 PMC1193112

[joa13892-bib-0009] Edman, K.A. & Reggiani, C. (1987) The sarcomere length‐tension relation determined in short segments of intact muscle fibres of the frog. The Journal of Physiology, 385, 709–732. 3498827 10.1113/jphysiol.1987.sp016516 PMC1192369

[joa13892-bib-0010] González‐Morales, N. , Xiao, Y.S. , Schilling, M.A. , Marescal, O. , Liao, K.A. & Schöck, F. (2019) Myofibril diameter is set by a finely tuned mechanism of protein oligomerization in drosophila. eLife, 8, e50496. 31746737 10.7554/eLife.50496 PMC6910826

[joa13892-bib-0011] Hart, P.E. (2009) How the Hough transform was invented. IEEE Signal Processing Magazine, 26, 18–22.

[joa13892-bib-0012] Henderson, C.A. , Gomez, C.G. , Novak, S.M. , Mi‐Mi, L. & Gregorio, C.C. (2017) Overview of the muscle cytoskeleton. Comprehensive Physiology, 7, 891–944. 28640448 10.1002/cphy.c160033 PMC5890934

[joa13892-bib-0013] Hu, L.Y.R. , Ackermann, M.A. & Kontrogianni‐Konstantopoulos, A. (2015) The sarcomeric M‐region: a molecular command center for diverse cellular processes. BioMed Research International, 2015, 1–25. 10.1155/2015/714197 PMC4413555 25961035

[joa13892-bib-0014] Isola, R. , Broccia, F. , Casti, A. , Loy, F. , Isola, M. & Vargiu, R. (2021) STZ‐diabetic rat heart maintains developed tension amplitude by increasing sarcomere length and crossbridge density. Experimental Physiology, 106, 1572–1586. 33977604 10.1113/EP089000 PMC8362044

[joa13892-bib-0015] Julian, F.J. & Morgan, D.L. (1979) The effect on tension of non‐uniform distribution of length changes applied to frog muscle fibres. The Journal of Physiology, 293, 379–392. 315465 10.1113/jphysiol.1979.sp012895 PMC1280719

[joa13892-bib-0016] Jungbluth, H. , Treves, S. , Zorzato, F. , Sarkozy, A. , Ochala, J. , Sewry, C. et al. (2018) Congenital myopathies: disorders of excitation–contraction coupling and muscle contraction. Nature Reviews Neurology, 14, 151–167. 29391587 10.1038/nrneurol.2017.191

[joa13892-bib-0017] Katti, P. , Hall, A.S. , Parry, H.A. , Ajayi, P.T. , Kim, Y. , Willingham, T.B. et al. (2022) Mitochondrial network configuration influences sarcomere and myosin filament structure in striated muscles. Nature Communications, 13, 6058. 10.1038/s41467-022-33678-y PMC9561657 36229433

[joa13892-bib-0018] Lange, S. , Ehler, E. & Gautel, M. (2006) From A to Z and back? Multicompartment proteins in the sarcomere. Trends in Cell Biology, 16, 11–18. 16337382 10.1016/j.tcb.2005.11.007

[joa13892-bib-0019] McMillan, J.D. & Eisenback, M.A. (2018) Transmission electron microscopy for analysis of mitochondria in mouse skeletal muscle. Bio‐Protocol, 8(10), e2455. 34285964 10.21769/BioProtoc.2455 PMC8275320

[joa13892-bib-0020] Mercier, L. , Böhm, J. , Fekonja, N. , Allio, G. , Lutz, Y. , Koch, M. et al. (2016) In vivo imaging of skeletal muscle in mice highlights muscle defects in a model of myotubular myopathy. Intravital, 5(1), e1168553. 28243519 10.1080/21659087.2016.1168553 PMC5226009

[joa13892-bib-0021] Picard, M. , White, K. & Turnbull, D.M. (2013) Mitochondrial morphology, topology, and membrane interactions in skeletal muscle: a quantitative three‐dimensional electron microscopy study. Journal of Applied Physiology, 114, 161–171. 23104694 10.1152/japplphysiol.01096.2012 PMC3544498

[joa13892-bib-0022] Pinotsis, N. , Abrusci, P. , Djinovic–Carugo, K. & Wilmanns, M. (2009) Terminal assembly of sarcomeric filaments by intermolecular beta‐sheet formation. Trends in Biochemical Sciences, 34, 33–39. 18996015 10.1016/j.tibs.2008.09.009

[joa13892-bib-0023] Ralston, E. , Swaim, B. , Czapiga, M. , Hwu, W.L. , Chien, Y.H. , Pittis, M.G. et al. (2008) Detection and imaging of non‐contractile inclusions and sarcomeric anomalies in skeletal muscle by second harmonic generation combined with two‐photon excited fluorescence. Journal of Structural Biology, 162, 500–508. 18468456 10.1016/j.jsb.2008.03.010 PMC2475676

[joa13892-bib-0024] Schönbauer, C. , Distler, J. , Jährling, N. , Radolf, M. , Dodt, H.U. , Frasch, M. et al. (2011) Spalt mediates an evolutionarily conserved switch to fibrillar muscle fate in insects. Nature, 479, 406–409. 22094701 10.1038/nature10559

[joa13892-bib-0025] Squire, J.M. (1997) Architecture and function in the muscle sarcomere. Current Opinion in Structural Biology, 7, 247–257. 9094325 10.1016/s0959-440x(97)80033-4

